# Kinase Insert Domain Receptor Q472H Pathogenic Germline Variant Impacts Melanoma Tumor Growth and Patient Treatment Outcomes

**DOI:** 10.3390/cancers16010018

**Published:** 2023-12-19

**Authors:** Milad Ibrahim, Irineu Illa-Bochaca, Faisal Fa’ak, Kelsey R. Monson, Robert Ferguson, Chen Lyu, Eleazar Vega-Saenz de Miera, Paul Johannet, Margaret Chou, Justin Mastroianni, Farbod Darvishian, Tomas Kirchhoff, Judy Zhong, Michelle Krogsgaard, Iman Osman

**Affiliations:** 1 Ronald O Perelman Department of Dermatology, NYU Grossman School of Medicine, New York, NY 10016, USA; miladadelmilad.ibrahim@nyulangone.org (M.I.); irineu.illabochaca@nyulangone.org (I.I.-B.); eleazar.vega-saenzdemiera@nyulangone.org (E.V.-S.d.M.);; 2 Department of Population Health, NYU Grossman School of Medicine, New York, NY 10016, USA; kelsey.monson@nyulangone.org (K.R.M.); robert.ferguson@nyulangone.org (R.F.); chen.lyu@nyulangone.org (C.L.); tomas.kirchhoff@nyulangone.org (T.K.); judy.zhong@nyulangone.org (J.Z.); 3 Department of Pathology, NYU Grossman School of Medicine, New York, NY 10016, USAmichelle.krogsgaard@nyulangone.org (M.K.); 4 Interdisciplinary Melanoma Cooperative Group, NYU Langone Health, 522 First Ave, New York, NY 10016, USA

**Keywords:** KDR, germline, melanoma, immune response, targeted therapy

## Abstract

**Simple Summary:**

Despite the improvement in melanoma treatment over the last decade, there is still a need for developing new treatment strategies that can achieve complete and durable remission. Herein, we provide evidence that the kinase domain receptor (KDR) pathogenic germline variant Q472H confers more angiogenic and proliferative tumors, a more immune-suppressive microenvironment, and more resistance to therapy. Data suggest that anti-angiogenic therapy might be beneficial in patients harboring this genotype.

**Abstract:**

Background: We previously reported a higher incidence of a pathogenic germline variant in the kinase insert domain receptor (KDR) in melanoma patients compared to the general population. Here, we dissect the impact of this genotype on melanoma tumor growth kinetics, tumor phenotype, and response to treatment with immune checkpoint inhibitors (ICIs) or targeted therapy. Methods: The KDR genotype was determined and the associations between the KDR Q472H variant (KDR-Var), angiogenesis, tumor immunophenotype, and response to MAPK inhibition or ICI treatment were examined. Melanoma B16 cell lines were transfected with KDR-Var or KDR wild type (KDR-WT), and the differences in tumor kinetics were evaluated. We also examined the impact of KDR-Var on the response of melanoma cells to a combination of VEGFR inhibition with MAPKi. Results: We identified the KDR-Var genotype in 81/489 (37%) patients, and it was associated with a more angiogenic (*p* = 0.003) and immune-suppressive tumor phenotype. KDR-Var was also associated with decreased PFS to MAPKi (*p* = 0.022) and a trend with worse PFS to anti-PD1 therapy (*p* = 0.06). KDR-Var B16 murine models had increased average tumor volume (*p* = 0.0027) and decreased CD45 tumor-infiltrating lymphocytes (*p* = 0.0282). The anti-VEGFR treatment Lenvatinib reduced the tumor size of KDR-Var murine tumors (*p* = 0.0159), and KDR-Var cells showed synergistic cytotoxicity to the combination of dabrafenib and lenvatinib. Conclusions: Our data demonstrate a role of germline KDR-Var in modulating melanoma behavior, including response to treatment. Our data also suggest that anti-angiogenic therapy might be beneficial in patients harboring this genotype, which needs to be tested in clinical trials.

## 1. Introduction

Specific germline genetic variants have been associated with increased cancer risk [1], but more recent data suggest a role for germline genetic variants in response to treatment and clinical outcome. A recent study supported the inclusion of germline analysis when considering patient stratification for treatment selection in cancer [2]. This has been supported by an analysis of more than 10,000 TCGA patients that identified 79 and 112 prognostic germline variants in individual and groups of cancer, respectively [3]. Another independent group reported a 7% frequency of pathogenic or likely pathogenic germline variants in 34,642 patients [4].

In melanoma, germline variants have been reported to be associated with thicker primary tumors [5], risk of toxicity to immune-checkpoint inhibition (ICI) [6], melanoma prognosis [7,8], and resistance to the combination of nivolumab and ipilimumab [9]. In addition, a germline variant in CDKN2A was reported to influence melanoma targeted therapy treatment outcomes [10]. Germline variants are also associated with response to treatment in melanoma, as shown by the association of the rs17388568 risk variant for allergy, colitis, and type 1 diabetes with anti-PD-1 response [6].

We previously reported a high incidence of the pathogenic germline variant Q472H in the kinase insert domain receptor (KDR) in melanoma, which was present in 35% of 1223 patients tested [11]. More recently, the same variant has been shown to be associated with increased angiogenesis and shorter survival in gliomas [12] and tyrosine kinase inhibitor resistance in non-small cell lung cancer [13].

Herein, we performed genomic, transcriptomic, and functional in vitro and in vivo analyses to define the impact of the germline variant KDR Q472H on melanoma biology and response to treatment.

## 2. Methods

### 2.1. Study Cohort

The study cohort included melanoma patients with available blood and tissues who were prospectively enrolled in an IRB-approved clinicopathological database at New York University (New York, NY, USA) Langone Health between 2002 and 2019 (IRB #10362) and received BRAF +/− MEK inhibition or ICI treatment. All patients provided written informed consent for the use of clinical data and/or biospecimens in research.

### 2.2. KDR Genotyping in Human Samples

Whole blood was collected from melanoma patients, and DNA was extracted as described previously [11]. Genotyping for the KDR variant Q472H was performed using the Applied Biosystems (Waltham, MA, USA) TaqMan Genotyping Master Mix (#4371353) and Assay (#4351379; rs1870377). KDR genotypes were assigned as major allele (wild type) homozygotes (TT), heterozygotes (TA), and minor allele homozygotes (AA).

### 2.3. Assessment of Microvessel Density (MVD)

Immunohistochemistry with anti-CD34 antibody (ab81289; dilution 1:500) was performed as described previously [11]. A board-certified pathologist (FD) blind to the patient’s KDR variant status scored MVD as follows. First, low magnification was used to select an intra-tumoral region with high vessel count. We deliberately avoided areas of high and obscuring melanin pigment deposition identified on low magnification. Then, at 200× magnification, the number of vessels was counted. Each independent CD34-positive vascular structure was counted as one vessel, whether circularly or linearly appearing, accounting for the perpendicular and horizontal cross sections, respectively. The secondary antibody used was a purple chromogen (ImmPACT VIP purple, Vector laboratories, SK-4605) to differentiate from the brown pigment. Dot plots were used to represent expression levels.

### 2.4. NanoString Gene Expression Analysis

RNA was isolated according to manufacturer protocols using the Qiagen RNAeasy DSP FFPE Kit. In cases of melanotic samples, the Zymo Research OneStep PCR Inhibitor Removal Kit was used. RNA was quantified with the Qubit 2.0 Fluorometer (Life Technologies, Carlsbad, CA, USA). Gene expression profiling was performed using the NanoString nCounter Human Immunology v2 Panel (CodeSet Only), and data were analyzed using nsolver^TM^ analysis software. Heatmaps were generated using R version 4.3.1 and the “pheatmap” package, which uses Euclidean distance as the similarity measure and clusters samples based on the ‘complete’ method [14]. PCA plots were generated using R version 4.3.1 and the “factoextra” package [15]. Clustering was performed via singular value decomposition.

### 2.5. TCGA Analysis

The TCGA Firehose Legacy melanoma dataset was analyzed in this study. Patients were classified into TA and AA combined as KDR-variant (KDR-Var) and TT as KDR-wild type (KDR-WT) according to their germline KDR status, and differential expression analyses were performed on cBioPortal using default analysis parameters. Pathways analysis was performed using Metascape and GO biological processes [16,17]. Somatic mutation analysis was performed by comparing the top 50 mutated genes in both groups using the chi square test. Oncoprints were generated using R version 4.3.1 and the “ComplexHeatmap” package [18].

### 2.6. Clinical Outcome Analyses

We assessed the relationship between KDR genotype and progression-free survival (PFS) in patients treated with MAPK inhibition or ICIs. MAPK inhibition included patients who received BRAF-inhibitors, BRAF + MEK-inhibitors, and MEK-inhibitors as a first line therapy. ICI-treated patients received anti-CTLA4, anti-PD1 antibodies, or the combination of both as first-line therapy, respectively. PFS was calculated as the number of months between treatment initiation and disease progression, last visit recorded, or death from disease. Kaplan–Meier curves were generated and compared using the log-rank test and MaxCombo test. Multivariable analyses included relevant clinicodemographic variables, such as age, gender, stage, and ECOG.

### 2.7. In Vitro Analysis of Cell Growth

Melanoma B16 cell lines [19] were transfected to express either KDR Q472Q WT or the KDR Q472H variant. For this purpose, a p*CMV3* containing the full coding sequence of the *VEGFR2* Q472Q wild type from SinoBiological (Wayne, PA, USA, Cat#HG10012-UT) was used as template. Next, a region of the *VEGF2* coding sequence containing the Q472H was produced using the following primers and site-directed mutagenesis by overlap extension [20]:

HVEGFR2_936F:TGGGCTGATGACCAAGAAGA NXT_HVEGFR2_v472H_R:GAGACAGCATGGCTTGGCTC, NXT_HVEGFR2_v472H_F:GAGCCAAGCCATGCTGTCTC, and HVEGFR2_2022R:CCAGCTTTCCTGTGATCGTG.

The product of this amplification was subcloned in p*Mini*T 2.0 (New England BioLab NEB, Ipswich, MA, USA), with both stands sequenced. A clone with the correct sequence was then restricted with *BstE* II/ *Afl* II and the 819BP band purified. The plasmid containing the VEGFR2 Q472Q was restricted with either *Afl* II and *Xba* I to generate a 2193 bp band or with *BstE* II HF and *Xba* I to generate a 7178 bp band. These three bands were purified then ligated with T4 ligase and transformed into NEB10ß competent cells, the resulting construct were digested, and three isolates with the proper restriction pattern were selected for sequencing. The VEGFR2 coding sequence was confirmed by double-strand sequencing via the deoxy sequencing method. All restriction enzymes were from NEB, and Platinum PFX (Invitrogen, Waltham, MA, USA) proofreader polymerase was used for all the amplifications. The mouse melanoma cell line B16-A2K^b^ was transfected with the VEGFR2 Q472Q or Q472H variant in p*CMV3* mammalian expression vector, and Lipofectamine 2000 (Invitrogen), the transfected cells, and individual colonies were selected with Hygromycin B gold (InvivoGen, San Diego, CA, USA). Cell growth for B16 cell lines, B16-A2Kb-VEGFQ472Q or B16-A2Kb-VEGFQ472H, KDR-WT, and KDR-Var, respectively, was characterized by in vitro colony assays.

A375 [21], SKMEL239 (MSKCC, New York), SKMEL192 [21], and SKMEL28 (ATCC) are KDR-WT melanoma cell lines. 501 mel [21] and WM2044 (Wistar Institute, Philadelphia, PA, USA) are KDR-V (homozygous) cell lines. 451Lu [21] and WM46 (Wistar Institute, Philadelphia, PA, USA) are KDR-Var (heterozygous) cell lines. Cells were treated with dose-escalated targeted therapies (Supplementary Materials Table S1). Cytotoxicity was assessed using the corrected absorbance from CellTiter 96^®^ AQueous One Solution Cell Proliferation Assay. The half maximal inhibitory concentration (IC_50_) values were calculated using dose response curves (Supplementary Materials Figure S1). Each experiment was replicated 4 times for the synergy assay. We used three equations to assess synergy: relative risk ratio (RRR) [22], the Bliss equation [23], and the combosyn combination index (CI) [24] (Supplementary Materials File S1). Three replicates were used for cell proliferation assays in response to different treatments. Independent *t*-tests were used to calculate significance.

### 2.8. In Vivo Analysis of KDR Q472H Melanoma Tumor Kinetics

All animal experiments were performed in accordance with NIH Institutional Animal Care and Use Committee Guidelines as well as with protocols approved by the Institutional Review Board at NYU Langone Health (IACUC ID #IA16-00618). Thirty-two 10-week-old CB6F1-Tg (HLA-A*0201/H2-Kb) A*0201 mice (Taconic) received a subcutaneous flank injection of either 2 × 10^5^ B16-A2Kb-VEGFQ472Q (KDR-WT) or 2 × 10^5^ B16-A2Kb-VEGF Q472H (KDR-Var) murine melanoma cells.

We first compared the growth kinetics of KDR-WT versus KDR-Var melanoma cells in murine models (*n* = 6 in each group). Tumors were allowed to grow for 29 days before undergoing resection. Tumor growth was measured with a caliper on days 0, 5, 9, 12, 14, 19, 21, 25, and 29. The total tumor volume was calculated using the formula V = length × width × height. Animals were sacrificed on day 29 after cell injection or when the tumors reached 10 mm, in accordance with institutional guidelines. Next, we analyzed the effect of lenvatinib (anti-angiogenic) therapy on the growth kinetics of KDR-WT versus KDR-Var tumors (*n* = 10 in each cohort). Within each cohort, mice were randomized to a control group (*n* = 5) receiving a placebo and a treatment group (*n* = 5) receiving Lenvatinib (40 mg/kg). We used the described experiment to determine the day of treatment initiation as the first time point of measurable tumor volume (day 12) and the day of treatment termination as the last time point before significant differences in tumor volumes between the KDR-WT and KDR-Var groups (day 21). Tumors were measured and calculated in the same manner described above. Animal group comparisons were performed using the Mann–Whitney test to compare two groups. Differential expression analysis was performed on the resected tumors using DE-Seq2 [25]. All functional analysis was carried out using g:Profiler [26]. Heatmaps were generated using R version 4.3.1 and the “Pheatmap” package, which uses Euclidean distance as the similarity measure and clusters samples based on the ‘complete’ method [14].

Flow cytometry was performed on tumors to assess the tumor-infiltrating lymphocyte (TIL) populations. Monoclonal antibodies included antibodies to CD45 (Biolegend, San Diego, CA, USA Cat. 103132, 1:100), CD3 (BioRad, Hercules, CA, USA Cat. MCA500PB, 1:100), CD4 (BD, Franklin Lakes, NJ, USA Cat. 553729, 1:100), and CD8 (BioLegend, San Diego, CA, USA, Cat. 100721, 1:100).

First, tumors were minced into small sections and then disaggregated using a plunger and a 70 μm cell strainer. Non-tumor cells were removed by washing with protein-free PBS. To assess cell viability, cells were incubated with aqua blue stain, washed, and then pelleted by centrifugation. For surface staining, cells were incubated with antibody in FACS buffer, washed, and then analyzed by flow cytometry. For intracellular staining, cells were incubated with FoxP3 Fix/Perm working solution, washed, and then incubated with anti-FoxP3 antibody (Thermo, East Windsor, NJ, USA cat. 17-5773-82, 1:100. After washing again, cells were resuspended in FACS buffer and analyzed using flow cytometry. The TIL values for different treatment groups were compared.

All statistical tests were performed using independent *t*-tests, *p* values were calculated with two-tailed tests, and the significant cutoff was <0.05.

## 3. Results

### 3.1. KDR-Var Patients Have More Proliferative, Immunosuppressive, and Angiogenic Tumors Compared to KDR-WT

Table 1 describes the baseline characteristics of the 489 patients studied. In total, 181 (37%) harbored the KDR Q472H variant, either as TA heterozygotes (*n* = 158) or AA homozygotes (*n* = 23), and 308 (63%) patients were major allele homozygotes (TT).

**Figure 1:**
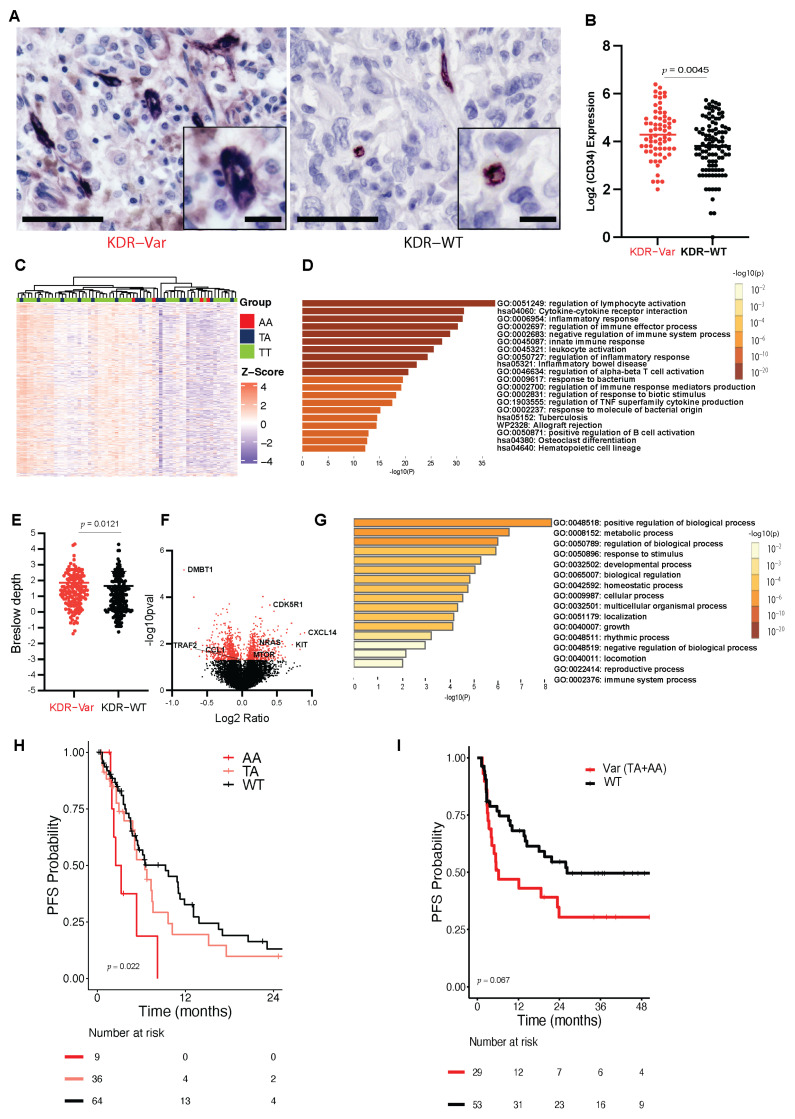
Genotypic and phenotypic analyses of melanomas derived from patients with germline variant KDR Q472H. (**A**) Representative images of CD34 expression in primary (**bottom**) and metastatic (**top**) melanoma tissue in KDR variant (**left**) and KDR WT (**right**). Scale bars are 100 μm and inserts are 50 μm. (**B**) Dot plot showing log2(CD34) expression of A (*p* = 0.0045). (**C**) Nanostring analysis of immunoregulatory gene expression. Differential gene expression analysis (N = 594 genes) illustrates distinct signatures in KDR-Var clusters and KDR-WT clusters in which the variant showed decreased expression (green) of genes associated with chemotactic activity, inflammatory responses, modulation of T cell activity, and antigen presentation (immunosuppression) as well as increased expression (red) of genes associated with cell growth and protein synthesis (proliferation). (**D**) Metascape pathway analysis of genes that are downregulated in KDR-Var. (**E**) TCGA dataset showing increased Breslow depth in the KDR-Var melanoma patients (*p* = 0.0121). (**F**) Volcano plot of DEGs between KDR-Var and KDR-WT in TCGA cohort analyzed by cBioPortal tool. Red color represents significant DEGs (*p* < 0.05) (**G**). Metascape pathway analysis of DEGS upregulated in the KDR-Var TCGA patients showing GO enriched biological processes. (**H**) Kaplan–Meier curve shows worse progression-free survival in AA (*n* = 9) homozygous and TA (*n* = 36) heterozygous KDR-Var patients compared to the KDR-WT TT (*n* = 64) variant when treated with first-line MAPK targeted therapies (*p* = 0.022). (**I**) Kaplan–Meier curve shows worse progression-free survival in KDR-Var patients (TA + AA combined, *n* = 29) compared to WT (*n* = 53) when treated with first-line PD-1-based therapy (*p* = 0.067). *p*-values were generated using log-rank test.

As a readout of microvessel density, CD34 was significantly increased in tumors from patients with the KDR-Var compared to KDR-WT (*n*= 172 (Supplementary Materials Table S2), *p* = 0.003, Figure 1A,B). NanoString nCounter Immunology Panel analysis was performed in a subset of 68 patients with sufficient metastatic tissue. Unsupervised clustering was performed using heatmaps (Figure 1C) and PCA plots (Supplementary Materials Figure S2A). In total, 60 genes were expressed at higher levels in KDR-WT patients and 4 were expressed at higher levels in KDR-Var patients (*p* < 0.05, Supplementary Materials File S2). Pathway analysis revealed that genes that are downregulated in KDR-var are enriched in the regulation of lymphocyte activation, cytokine–cytokine receptor interaction, and inflammatory response pathways, among others (Figure 1D). Genes expressed highly in KDR-WT tumors comprised a total of 22 chemokines, interleukins, and interferons, including *LAMP3*, which is a marker of tertiary lymphoid structures (TLS, *p* = 0.010), and *HAVCR2*, which is an immune checkpoint regulator and is also known as *TIM-3* (*p* = 0.040). Downregulation of genes associated with chemotactic activity (e.g., *CCL8*, *p* = 0.004), inflammatory responses (*LILRB1*, *p* = 0.0079), modulation of T cell activity (*IL-7*, *p* = 0.019), and antigen presentation (*LILRB4* *p* = 0.015) was observed in tumors from KDR-Var patients. In contrast, *STAT3* and *RPL19* were both overexpressed in the KDR-Var tumors (*p* = 0.037, 0.019, respectively). Expression of these two genes is known to be associated with cell growth and protein synthesis.

In the TCGA dataset, KDR-Var was found in 188/451 patients (39.9%), a similar frequency to that observed in our cohort. KDR-Var was significantly associated with increased Breslow depth, which is an independent marker for worse prognosis in primary melanoma [27] (*p =* 0.0121, Figure 1E). There was a total of 1175 differentially expressed genes (DEGs) when comparing TCGA KDR-Var to KDR-WT using the cBioPortal webtool [16]. There were 524 upregulated and 651 downregulated genes (Figure 1F). KDR-Var upregulated genes were enriched in growth (*p* = 0.0001) and immune system pathways (*p* = 0.01), as shown by GO biological processes (Figure 1G), and included *KIT*, *NRAS*, *MTOR*, *CRK*, and *PDPK1*, all reported to be oncogenic regulators of cell proliferation. Downregulated genes also showed enrichment immune process (Supplementary Materials Figure S2B). KDR-Var was significantly associated with decreased mutations in *COL2A1* (19% of KDR-Var vs. 30% of KDR WT, *p* = 0.01, Supplementary Materials, Figure S3). There was no difference in KDR expression nor KDR somatic mutations between the two groups.

### 3.2. KDR Q472H Variant Is Associated with Worse Response to Treatment

A total of 343 (209 KDR-WT and 134 KDR-Var) patients received either BRAF +/− MEK targeted therapy or ICIs. Among patients treated with targeted therapy (*n* = 109), multivariable analysis showed that the KDR-WT group had a PFS of 18.97 months compared to 14.66 months in patients who were KDR-Var heterozygous and 6.87 months in those who were KDR-Var homozygous (*p* = 0.022, Figure 1H). Among patients treated with ICIs, PFS was comparable in both groups. However, there was a trend toward worse PFS in patients who received anti-PD1-based therapies as a first-line treatment. PFS was 26.3 months in KDR-WT and 5.88 months in KDR-Var patients (*n* = 82 Log rank *p* = 0.067, MaxCombo *p* = 0.0987, Figure 1I). A similar trend was observed in overall survival of our cohort (10.7 months KDR-Var vs. 17.09 months KDR-WT, *p* = 0.081, Supplementary Materials, Figure S4).

### 3.3. In Vitro and In Vivo Melanoma Models Induced with Germline Variant KDR Q472H Have Higher Proliferation Rates and more Immunosuppressant and Pro-Angiogenic Tumors

**Figure 2:**
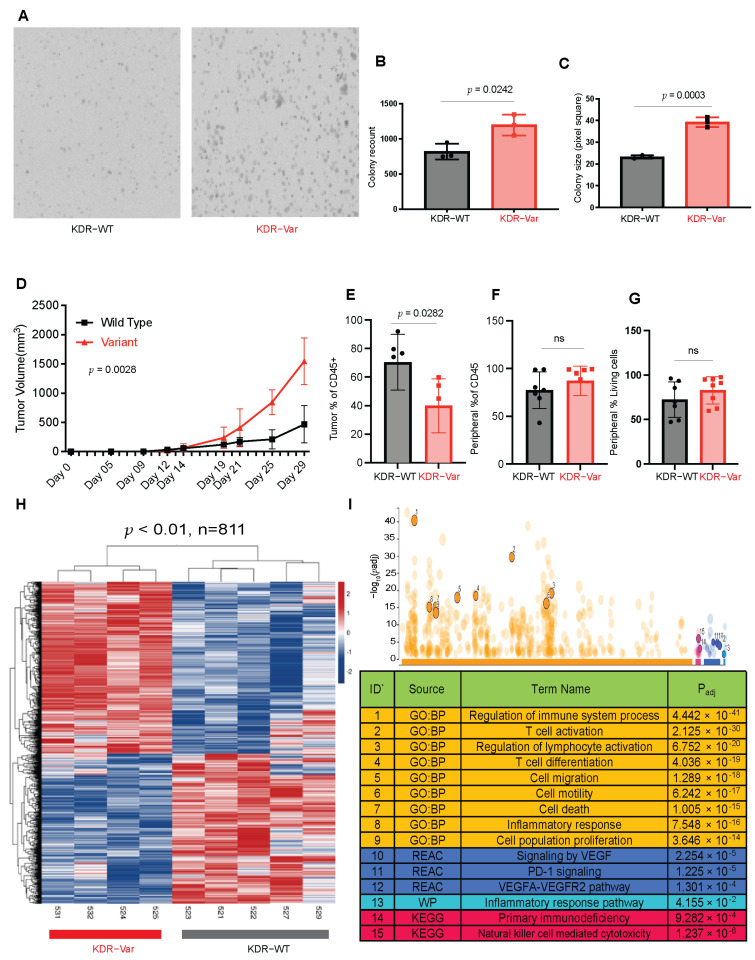
Analysis of cell line growth, tumor kinetics, and microenvironments in B16 animal models induced with germline variant KDR Q472H. (**A**–**C**). Colony assay comparing B16 cell lines induced with KDR WT or KDR Q472H showing increased KDR-Var colony count (*p* = 0.0242) and colony size (*p* = 0.0003). (**D**) Tumor growth curve shows the average tumor volume for mice induced with the KDR variant Q472H at day 29 is greater than the average tumor volume for mice without the KDR variant (*p* = 0.0027). (**E**–**G**). Box blots showing significant reduction in CD45+ tumor-infiltrating lymphocytes in the KDR-Var compared to the KDR-WT (*p* = 0.0282), while no difference is observed in the peripheral blood. (**H**) Heat map from RNA-Seq analysis of tumors resected from mice induced with KDR-V B16 cells (*n* = 4) or KDR WT (*n* = 5) shows distinct clusters of the two groups comparing DEGs between two groups at *p* < 0.01. (**I**) g:Profiler pathway analysis of RNA-seq DEGs comparing KDR-Var to KDR-WT mice tumors showing enriched key pathways and cellular processes like regulation of immune system response, cell proliferation, and signaling by VEGF.

KDR-Var B16 cells showed higher colony count (*p* = 0.0242) and bigger colony size compared to KDR-WT (*p* = 0.0003) cells (Figure 2A–C). Mice injected with KDR-Var expressing tumor cells showed increased average tumor volume (ATV) on day 29 (endpoint) (ATV = 1546 mm^3^) compared to the KDR-WT induced mice (ATV = 468 mm^3^, *p* = 0.0027, Figure 2D). KDR-Var tumors also showed decreased CD45 tumor-infiltrating lymphocytes (TILs) compared to KDR-WT tumors (*p* = 0.0282), but no difference was observed in peripheral blood (Figure 2E–G).

RNA-Seq analysis of tumors isolated from mice showed distinct clusters in KDR-Var tumors compared to WT tumors as shown by heatmaps (Figure 2H). There was a total of 1120 DEGs at a *p* < 0.05 and an FDR value below 0.05. These DEGs were enriched in regulation of immune response, T cell activation and differentiation, inflammatory response, cell proliferation, and cell death biological processes, as well as VEGF, PD-1 and inflammatory process pathways (Figure 2I). We next filtered the DEGs to include only −2 > Fold change (FC) > 2, which resulted in a total of 397 DEGs, 270 of which were up- and 127 downregulated in KDR-Var tumors, respectively (Supplementary Materials File S3). Functional analysis of the 270 upregulated DEGs showed that these genes remain enriched in angiogenesis, immune response, and cell proliferation pathways. Gene ontology identified 47 DEGs involved in immune response, 34 in inflammatory response, 16 in cell proliferation, 14 in positive regulation of cell migration, 11 in positive regulation of ERK cascade, 10 in angiogenesis, and 9 in positive regulation of the MAPK cascade (Supplementary Materials File S4). Of note, *LAG3* was overexpressed in KDR-Var tumors (FC = 2.41, *p* = 6.16 10^−5^, FDR = 0.003), as well as Gimap*3* (FC = 4.52, *p* = 04.76 10^−7^, FDR = 8.71 10^−5^)*, Tigit* (FC = 3.6, *p* = 5.62 10^−5^, FDR = 0.003), *Lax1* (FC = 5.82, *p* = 1.92 10^−7^, FDR = 4.19 10^−5^), and *Slfn1* (FC = 2.4, *p* = 1.46 10^−4^, FDR = 0.006). all of which are known to be downregulators of the immune response. There were downregulated genes in the KDR-Var that negatively affect cell proliferation (*CD9* FC = −4.32, *p* = 7.42 10^−10^, FDR = 5.87 10^−7^, *Cav1* FC = −1.658621788, *p* = 3.70 10^−6^, FDR = 4.37 10^−4^, *Cav2* FC = −3.8, *p* = 2.37 10^−9^, FDR = 1.48 10^−6^, Igfbp3 FC = −2.18, *p* = 2.28 10^−4^, FDR = 0.008).

**Figure 3:**
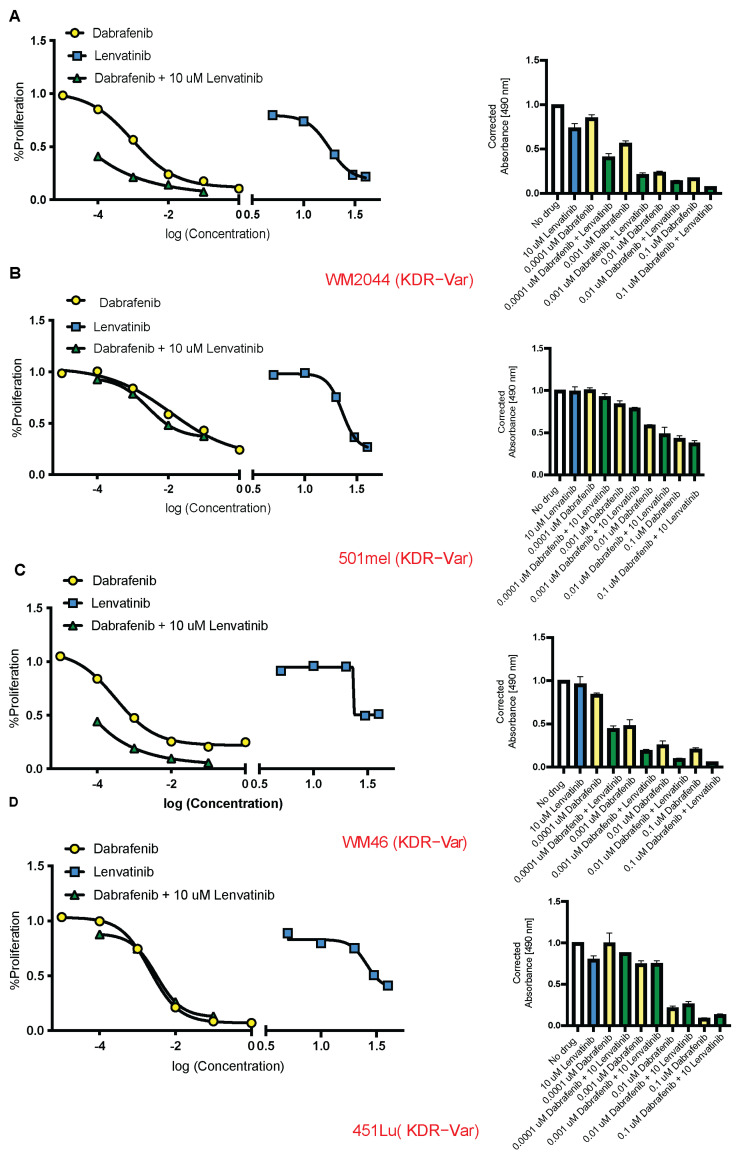
Germline variant KDR Q472H cell lines display synergistic cytotoxicity when treated with VEGFR + BRAF inhibitors. Difference between the observed and expected inhibition of cell growth when combining Lenvatinib 10 μM with varying doses of Dabrafenib. Expected inhibition was calculated with the Combosyn combination index (*n* = 4). (**A**) WM2044 (AA). (**B**) 501MEL (AA). (**C**) WM46 (TA). (**D**) 451LU (TA). Yellow, blue and green represent Dabrafenib, Lenvatinib and a combination of both, respectively.

**Figure 4:**
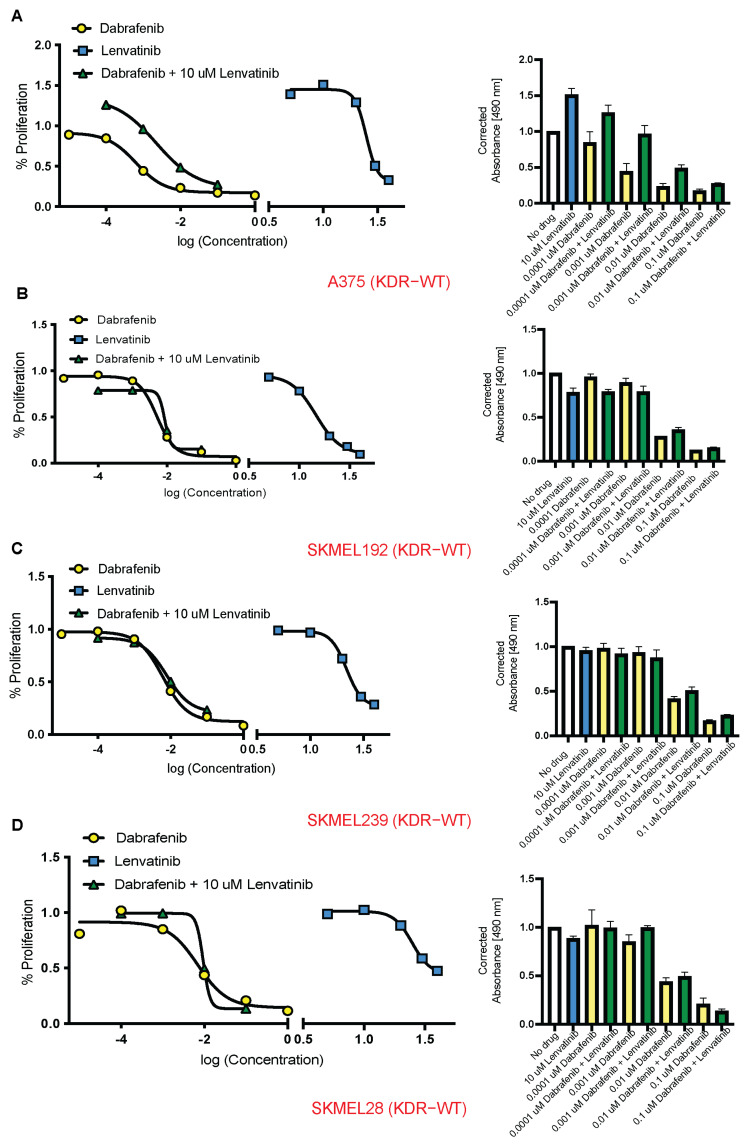
KDR-WT cell lines do not display synergistic cytotoxicity when treated with VEGFR + BRAF inhibitors. Difference between the observed and expected inhibition of cell growth when combining Lenvatinib 10 μM with varying doses of Dabrafenib. Expected inhibition was calculated with Combosyn combination index (*n* = 4). (**A**) A375. (**B**) SKMEL192. (**C**) SKMEL239. (**D**) SKMEL28. Yellow, blue and green represent Dabrafenib, Lenvatinib and a combination of both, respectively.

In the homozygous KDR-Var cell lines, WM2044 and 501 mel, the combination of dabrafenib + 10 μM lenvatinib, yielded synergistic inhibition at every dose level of dabrafenib (WM2044: 0.64, 0.5, 0.79, and 0.57; 501 mel: 0.92, 0.94, 0.82, and 0.878 RRR for 0.0001, 0.001, 0.01, and 0.1 μM dabrafenib, respectively) (Figure 3A,B). In the heterozygous KDR-Var cell line, WM46, the combination yielded synergistic inhibition at every dose level of dabrafenib (0.54, 0.41, 0.39, and 0.28 RRR for 0.0001, 0.001, 0.01, and 0.1 μM dabrafenib, respectively) (Figure 3C). The combination yielded synergetic inhibition at 0.0001μM in the heterozygous KDR-Var cell line 451Lu (1.1, 1.25, 1.53, and 1.91 RRR for 0.0001, 0.001, 0.01, and 0.1 μM dabrafenib, respectively) (Figure 3D). In the KDR-WT cell lines, A375 and SKMEL192, there was no synergy found at any dabrafenib dose (A375: 1.01, 1.44, 1.38, and 1.055; SKMEL192: 1.05, 1.13, 1.62, and 1.58 RRR for 0.0001, 0.001, 0.01, and 0.1 μM dabrafenib, respectively) (Figure 4A,B). In the KDR-WT cell lines, SKMEL239 and SKMEL28, the combination yielded synergistic inhibition at one dabrafenib dose only (SKMEL239: 0.97, 1, 1.28, and 1.44; SKMEL28: 1.04, 1.3, 1.25, and 0.71 RRR for 0.0001, 0.001, 0.01, and 0.1 μM dabrafenib, respectively) (Figure 4B–D). The median RRR for KDR-Var (0.81, *n* = 12) was significantly lower than the median RRR for KDR-WT (1.19, *n* = 12) (*p* = 0.007).

**Figure 5:**
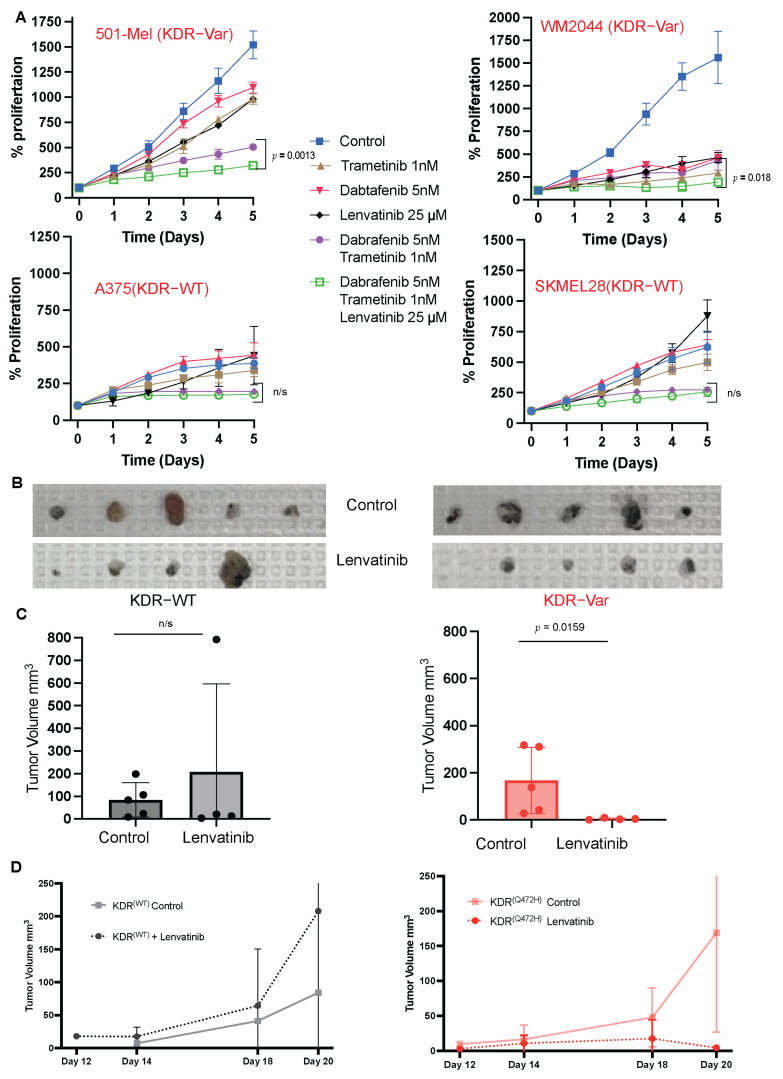
Germline variant KDR Q472H cell lines and murine models respond to Lenvatinib treatment. (**A**) Growth curves of two KDR homozygous variant and two KDR wild type melanoma cell lines showing decreased proliferation upon the addition of Lenvatinib (VEGFR inhibitor) to Dabrafenib (BRAF inhibitor) and Trametinib (MEK inhibitor) combination in the KDR-Var and not the WT. (**B**) Representative images of tumors resected from B16 mice post-treatment with control (**top**) or the anti-VEGFR, Lenvatinib (**bottom**). (**C**) Bar graphs of tumor volume at endpoint in KDR WT (**left**) and KDR variant (**right**) B16 mouse models post-treatment. (**D**) Growth curves of tumors with or without Lenvatinib in KDR WT (**left**) or KDR variant (**right**).

The addition of lenvatinib to the current standard BRAF + MEK targeted therapy dabrafenib plus trametinib reduced the proliferation of KDR-V cell lines compared to a combination of both treatments. For 501 mel, the average % proliferation after 5 days (*n* = 3) for triple therapy was 322%, compared to 505% for the combination of dabrafenib plus trametinib (*p* = 0.0013). For WM2044 (*n* = 3), the triple therapy average was 192%, compared to 430% for the combination of dabrafenib plus trametinib (*p* = 0.018). There was no significant reduction in the proliferation of KDR-WT cell lines upon the addition of lenvatinib to BRAF + MEK combination. For A375 (*n* = 3), the average proliferation after 5 days was 178% for triple therapy and 195% for the combination of dabrafenib plus trametinib (*p* = 0.12). For SKMEL28 (*n* = 3), 257% and 274% were the observed average % proliferation for triple therapy and dabrafenib plus trametinib combination, respectively (*p* = 0.46) (Figure 5A). Of note, the average % proliferation upon treating the cells with the combination of dabrafenib + trametinib was observed to be higher in KDR-Var cell lines compared to KDR-WT cell lines, indicating resistance of KDR-Var cell lines to this combination of treatment. Finally, we tested the effect of lenvatinib on KDR-V murine tumors and compared the effects to KDR WT tumors. Lenvatinib treatment reduced the size of KDR-V induced murine tumors compared to the untreated group (*p* = 0.0159). (Figure 5B–D).

## 4. Discussion

We previously reported on the high incidence of germline variant KDR Q472H in a cohort of more than 1000 melanoma patients and its association with increased microvessel density in human tissues [11]. In this study, we expanded our studies to further dissect the impact of this genotype on melanoma tumor phenotype and response to treatment. Our data revealed several important points. First, melanoma patients who harbor the germline variant KDR Q472H have more aggressive tumor biology, as shown by a more proliferative, immunosuppressive phenotype, in addition to being more angiogenic compared to KDR-WT tumors. Second, the KDR Q472H variant is associated with significantly worse response to targeted therapy and a trend with worse response to anti-PD-1 antibodies. Third, our preclinical data using both in vitro and in vivo analyses of melanoma B16 cell lines transfected with the KDR-V or wild type (WT) gene supported the evidence suggested by our clinical correlative data. Taken together, our data provide evidence that germline variants, in this case KDR Q472H, might interact with somatic tumor driver mutations to further promote tumor progression and could also support novel therapeutic modalities.

Angiogenesis can have direct effects on immune cell adhesion and extravasation, and the abnormal tumor vasculature also indirectly antagonizes the effectiveness of cancer immunotherapy by promoting TME-mediated immune suppression [28,29]. Our data revealed that KDR Q472H can alter an anti-tumor immune response as a result of increased angiogenesis. Genes that were downregulated in KDR-V tumors included the T-bet transcription factor, which has been shown to play a critical role in response to ICIs [30] and is an essential master regulator of gene expression that guides type 1 immune helper differentiation in naive T cells and other immune cells. Tbet also plays a specific role in the differentiation of cytotoxic T cells and activation of natural killer cells [31]. Similarly, there were significantly lower levels of critical immune response regulators such as *IFN*g, which induces or enhances MHC class I antigen presentation, in KDR-V compared with KDR-WT tumors [32]. Downregulation of IFNg may result in a significantly decreased MHC class I antigen presentation, allowing immune evasion. In addition, expression of key cytokine genes was lower in KDR-V compared with KDR-WT tumors, such as interleukin-2, which is known for its well-characterized role as a T-cell growth factor leading to the growth and expansion of T-cells, particularly CD8+ T-cells, and documented anti-tumor efficacy in advanced renal cancer and melanoma [33].

Differentially expressed immune checkpoints in KDR-V tumors suggest an immune-resistant tumor microenvironment. *LAG3* is a checkpoint that suppresses T cell activation and cytokine secretion, impacting different lymphocyte phenotypes [34]. Similarly, cancer cells use the *TIGIT* checkpoint as an immune evasion mechanism, downregulating antigen presentation, inhibiting malignant cell killing by T cell and natural killer (NK) cells, and enhancing the immune suppressive activity of regulatory T cells [35]. This study provides evidence that the KDR Q472H germline variant drives a more aggressive tumor phenotype by promoting the proliferation of human melanoma tumor cell lines as shown and independently validated in TCGA data and murine models. Moreover, the association of KDR-Var with an increased Breslow depth [36] adds to the evidence of its more aggressive phenotype.

The combination of ICIs and VEGFR inhibitors has recently been FDA-approved and is now being used in patients with kidney, liver, lung, and endometrial cancer [37]. Moreover, there are open clinical trials that are testing this combination in melanoma (NCT04356729). Also, very recently, it was shown that such a combination can augment melanoma anti-tumor activity [38]. Taken together, our data suggest that the KDR Q472H germline variant may identify a subset of melanoma patients that can benefit from this combination. Our data show that KDR-Var melanoma patients who receive targeted therapy have worse survival outcomes. The KDR-Var cell lines used in this study did not show different response to lenvatinib and dabrafenib as single therapies in the doses tested in this study. However, KDR-Var and not WT showed benefit from the addition of lenvatinib to BRAF inhibitors alone or in combination with MEK inhibition. The same effect was shown in murine models. However, there is a tumor in the KDR-WT murine group that appears to be an outlier in this group, which could be due to biological variance or technical errors [39].

Of note, it will also be interesting to associate germline variation in KDR with melanoma susceptibility. While this was not the focus of our studies, there are other potential germline variants that were identified in the sequencing efforts in the general population, and it will be interesting to link this with melanoma susceptibility. While none of the associations of KDR var have been found in the previous extensive GWAS efforts in melanoma, it is unclear if KDR plays a role in melanoma risk. However, given the less common frequency of these variants, future studies should investigate the effect of these infrequent alleles in a large melanoma cohort.

## 5. Conclusions

In sum, melanoma tumors from KDR-V patients possess an immune altered tumor microenvironment as well as a hyper-vasculature and hyper-proliferative phenotype. Tumors from KDR-V patients are associated with worse treatment outcomes and can be more sensitive to anti-VEGF treatment. This study highlights the importance of a germline variant analysis in melanoma for better stratification and treatment selection. In the future, more studies to validate the use of anti-VEGFR treatment in combination with targeted or immunotherapy in this subset of patients should be prioritized.

## Figures and Tables

**Table 1:** Characteristics of patients enrolled in the IMCG and characterized for the KDR-V Q472H.

	AA	TA	TT	*p*-Value
*n*	23	158	308	
Age (mean (SD))	59.18 (16.22)	61.21 (15.68)	60.04 (15.91)	0.701
Gender = M (%)	10 (43.5)	91 (57.6)	198 (64.3)	0.077
Stage (%)				0.278
I	5 (21.7)	18 (11.4)	32 (10.4)	
II	2 (8.7)	23 (14.6)	59 (19.2)	
III	4 (17.4)	56 (35.4)	96 (31.2)	
IV	11 (47.8)	55 (34.8)	101 (32.8)	
NA	1 (4.3)	6 (3.8)	20 (6.5)	
Follow up (mean (SD))	63.04 (67.74)	59.58 (51.18)	60.87 (73.05)	0.965
Treatment (%)				0.149
Targeted therapy	9 (39.1)	41 (25.9)	81 (26.3)	
Immunotherapy	12 (52.1)	74 (46.8)	135 (43.8)	
Chemotherapy	1 (4.3)	2 (1.3)	10 (3.2)	
Interferon	0 (0.0)	0 (0.0)	1 (0.3)	
Radiation therapy	0 (0.0)	2 (1.3)	9 (2.9)	
Surgery	1 (4.3)	36 (22.8)	67 (21.8)	
Unknown	0 (0.0)	3 (1.89)	5 (1.62)	

## Data Availability

The data generated or analyzed during this study are included here. Nanostring and RNA-Seq data are submitted in the Supplementary Materials. Additional data will be made available from the corresponding author on reasonable request.

## Supplementary Materials

The following supporting information can be downloaded at: https://www.mdpi.com/article/10.3390/cancers16010018/s1, Figure S1: IC50s; Figure S2: PCA; Figure S3: Oncoprints; Figure S4: survival analysis; Table S1: Treatment Groups; Table S2: IHC patients’ characteristics. File S1: Synergy raw data equations; File S2: Nanostring analysis; File S3: RNA-Seq; File S4 Pathway analysis.

## Abbreviations

KDR-V	Kinase insert domain receptor Q472H
KDR-WT	Kinase insert domain receptor Q472Q
ICI	Immune checkpoint inhibitors
MVD	Microvessel density
PFS	Progression-free survival
TIL	Tumor-infiltrating lymphocytes
FC	Fold change
NK	Natural killer
