# Supplementary material for: Kinase Insert Domain Receptor Q472H Pathogenic Germline Variant Impacts Melanoma Tumor Growth and Patient Treatment Outcomes
Source: Cancers (Basel). 2023 Dec 19;16(1):18. doi: 10.3390/cancers16010018 (PMC10778134; doi:10.3390/cancers16010018)
Supplement: Supplementary file 1 [file cancers-16-00018-s001.zip › Supplementary/Table S1 treatment groups.pdf]

Supplementary Table S1 melanoma cell lines treatment groups used for synergy assays

Treatment	Anti-VEGFR (Lenvatinib)	Anti-MAPK (Darbrafenib)
Control	-	-
Anti-VEGFR monotherapy	10µM	-
low dose anti-MAPK monotherapy	-	0.0001µM
low dose combination therapy	10µM	0.0001µM
medium-low dose anti-MAPK monotherapy	-	0.001µM
medium-low dose combination therapy	10µM	0.001µM
medium-high dose anti-MAPK monotherapy	-	0.01µM
medium-high dose combination therapy	10µM	0.01µM
high dose anti-MAPK monotherapy	-	0.1µM
high dose combination therapy	10µM	0.1µM
