# Supplementary material for: Kinase Insert Domain Receptor Q472H Pathogenic Germline Variant Impacts Melanoma Tumor Growth and Patient Treatment Outcomes
Source: Cancers (Basel). 2023 Dec 19;16(1):18. doi: 10.3390/cancers16010018 (PMC10778134; doi:10.3390/cancers16010018)
Supplement: Supplementary file 1 [file cancers-16-00018-s001.zip › Supplementary/Table S2 IHC patients.pdf]

Supplementary Table S2: CD34 IHC patients characteristics

	**AA**	**TA**	**TT**	
n	6	60	106	p-val
Age (mean (SD)	59.16 (21)	59.3 (17)	56.35 (17.46)	0.5453
Gender = M (%)	3 (50)	36 (60)	64 (61.3)	0.8803
Stage (%)				0.7245
I	2 (33.3)	12 (20)	21 (19.8)	
II	1 (16.6)	19 (31.6)	40 (37.7)	
III	3 (50)	29 (48.3)	41 (38.6)	
IV			2 (1.88)	
NA			2 (1.88)	
