# Supplementary figures and images for: Kinase Insert Domain Receptor Q472H Pathogenic Germline Variant Impacts Melanoma Tumor Growth and Patient Treatment Outcomes
Source: Cancers (Basel). 2023 Dec 19;16(1):18. doi: 10.3390/cancers16010018 (PMC10778134; doi:10.3390/cancers16010018)

## Slide 1
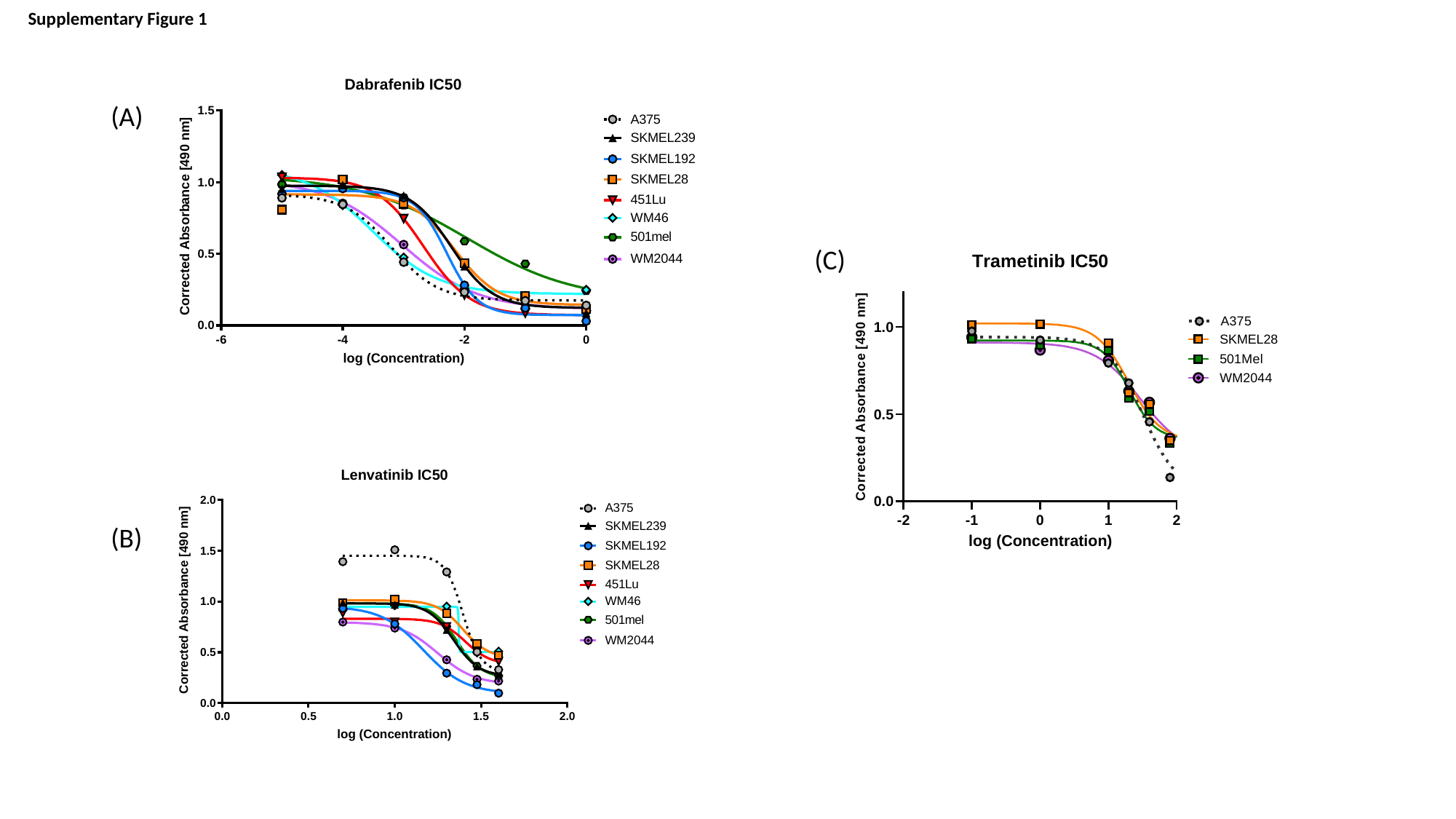

Supplementary Figure 1
(A)
(C)
(B)

Supplement: Supplementary file 1 [file cancers-16-00018-s001.zip › Supplementary/Figure_S1_IC50.pptx]

## Slide 1
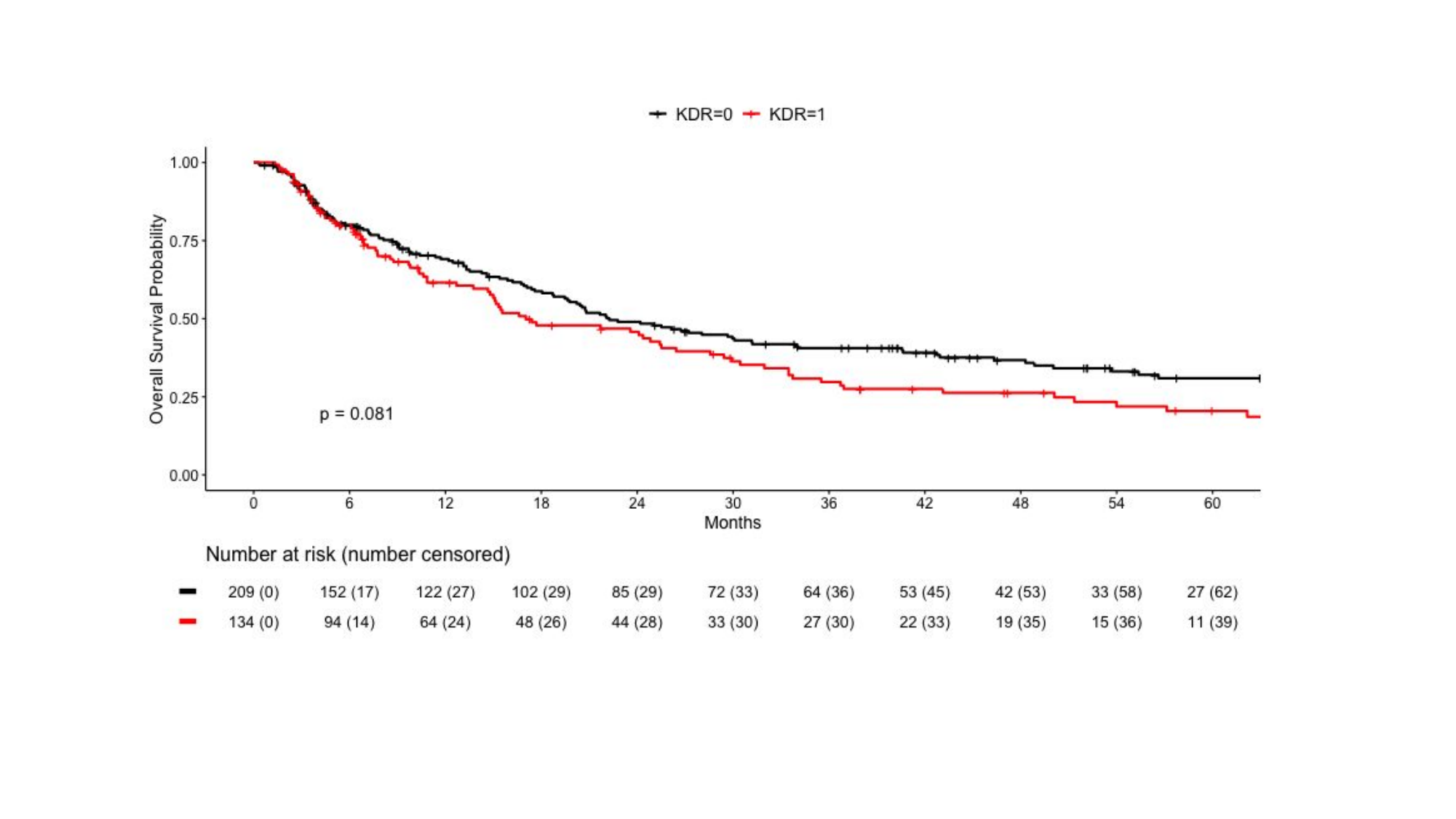

Supplement: Supplementary file 1 [file cancers-16-00018-s001.zip › Supplementary/Figure_S4_Km.pptx]

## Slide 1
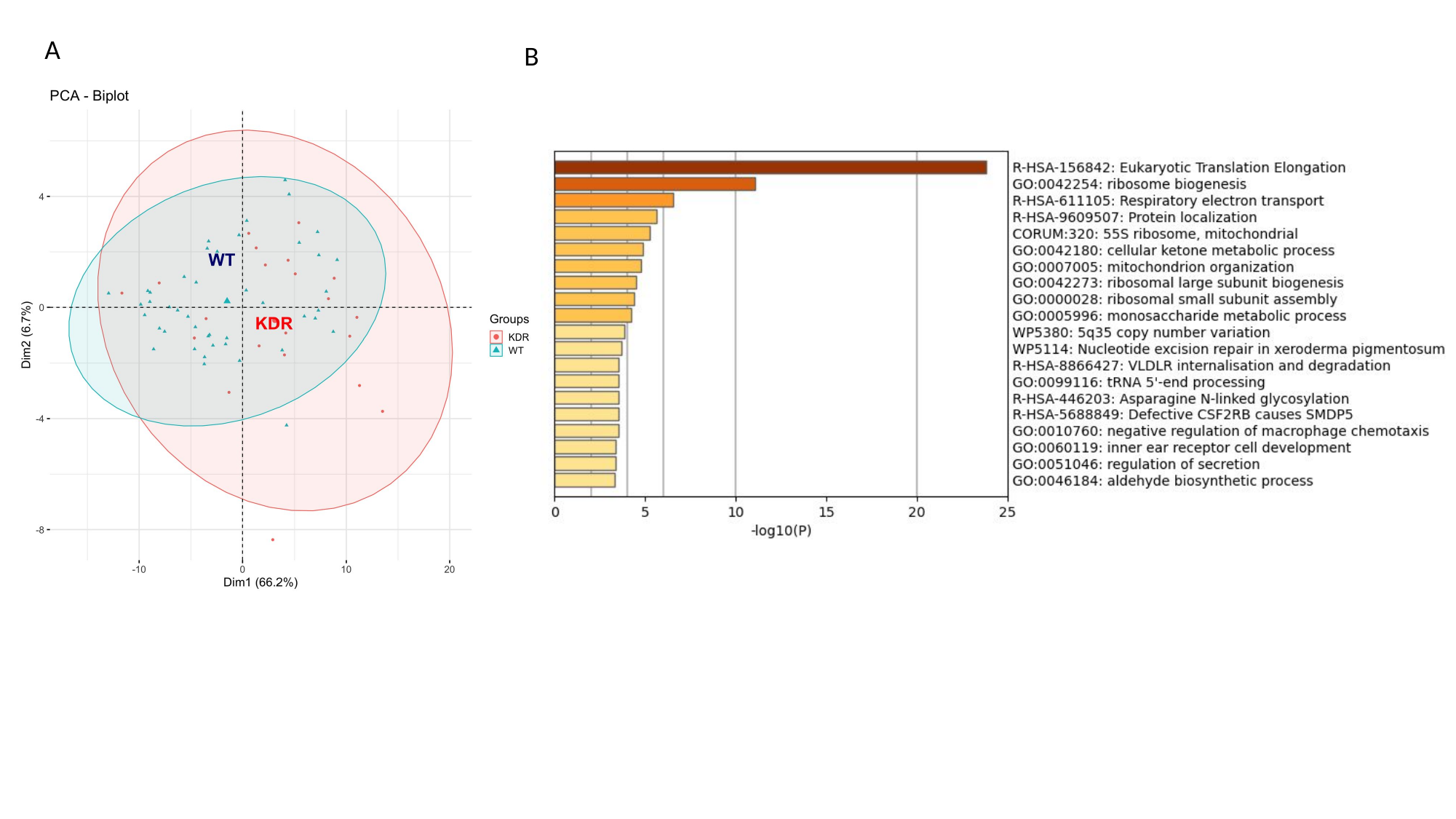

A
B

Supplement: Supplementary file 1 [file cancers-16-00018-s001.zip › Supplementary/FigureS2_PCA.pptx]

## Slide 1
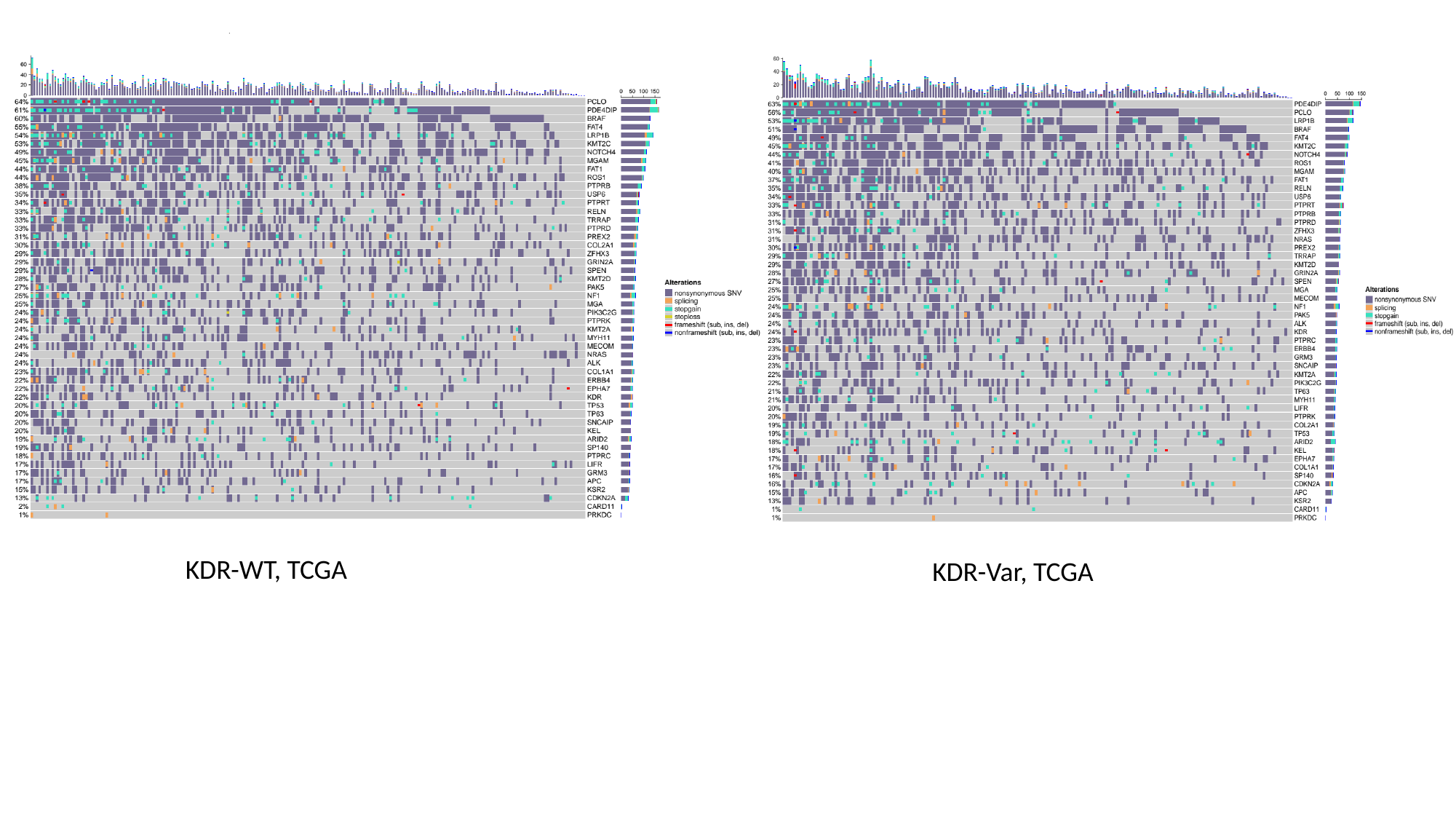

KDR-WT, TCGA
KDR-Var, TCGA

Supplement: Supplementary file 1 [file cancers-16-00018-s001.zip › Supplementary/FigureS3_Oncoprints.pptx]
